# A nested cohort 5-year Canadian surveillance of Gram-negative antimicrobial resistance for optimized antimicrobial therapy

**DOI:** 10.1038/s41598-023-40012-z

**Published:** 2023-08-29

**Authors:** Joseph Blondeau, Marthe Kenny Charles, Vivian Loo, Heather Adam, Marcela Gonzalez Del Vecchio, Christiane Ghakis, Emma O’Callaghan, Radwan El Ali

**Affiliations:** 1 Clinical Microbiology, Royal University Hospital and the Saskatchewan Health Authority, and the Departments of Pathology and Laboratory Medicine, Microbiology, Immunology and Biochemistry, and Ophthalmology, University of Saskatchewan, Saskatoon, SK Canada; 2 Department of Pathology and Laboratory Medicine, University of British Columbia, Vancouver General Hospital, Vancouver, BC Canada; 3 Division of Infectious Diseases, Department of Medicine, McGill University and McGill University Health Centre, Montreal, Canada; 4 Department of Medical Microbiology and Infectious Diseases, Max Rady College of Medicine, University of Manitoba and Diagnostic Services, Shared Health, Winnipeg, MB Canada; 5 Global Medical Affairs, Merck & Co, Inc., Kenilworth, NJ USA; 6 Medical and Scientific Affairs, Merck Canada Inc., Kirkland, QC Canada; 7 Formerly affiliated With Merck Canada Inc., Kirkland, QC Canada

**Keywords:** Microbiology, Diseases, Health care, Pathogenesis

## Abstract

We analyzed 5 years (2016–2020) of nested Canadian data from the Study for Monitoring Antimicrobial Resistance Trends (SMART) to identify pathogen predominance and antimicrobial resistance (AMR) patterns of adult Gram-negative infections in Canadian health care and to complement other public surveillance programs and studies in Canada. A total of 6853 isolates were analyzed from medical (44%), surgical (18%), intensive care (22%) and emergency units (15%) and from respiratory tract (36%), intra-abdominal (25%), urinary tract (24%) and bloodstream (15%) infections. Overall, *E. coli* (36%), *P. aeruginosa* (18%) and *K. pneumoniae* (12%) were the most frequent isolates and *P. aeruginosa* was the most common respiratory pathogen. 18% of Enterobacterales species were ESBL positive. Collective susceptibility profiles showed that *P. aeruginosa* isolates were highly susceptible (> 95%) to ceftolozane/tazobactam and colistin, though markedly less susceptible (58–74%) to other antimicrobials tested. Multi-drug resistance (MDR) was present in 10% of *P. aeruginosa* isolates and was more frequent in those from respiratory infections and from ICU than non-ICU locations. Of *P. aeruginosa* isolates that were resistant to combinations of ceftazidime, piperacillin/tazobactam and meropenem, 73–96% were susceptible to ceftolozane/tazobactam over the period of the study. These national data can now be combined with clinical prediction rules and genomic data to enable expert antimicrobial stewardship applications and guide treatment policies to optimize adult patient care.

## Introduction

Optimization of antimicrobial therapy requires the selection of appropriate treatment that must be administered as quickly as possible^1^. This is particularly challenging in the hospital setting, where antimicrobial resistance (AMR) is a major barrier to effective treatment selection^2^. To address this critical problem, Canada has developed a Federal Action Plan^3^, a National Action Plan for Stewardship^4^, has a Pan-Canadian Framework for Action^5^ and rigorous professional guidelines on AMR^6^, while the Canadian Institutes of Health Research have highlighted a priority program to define the incidence, epidemiology, consequences, costs and solutions to this growing epidemic^7^.

Gram-negative organisms dominate the World Health Organization Priority Pathogens List^8^ and the CDC 2019 Antibiotic Resistance Threats Report^9^ and represent a particular challenge by virtue of their opportunistic predilection for at-risk individuals, rapid colonization and nosocomial spread, high morbidity and mortality, rapid development of AMR and limited treatment options^10, 11^. *Pseudomonas aeruginosa* and Enterobacterales species exhibit both chromosomal antimicrobial resistance related to restricted outer membrane permeability, efflux systems and antibiotic-inactivating enzymes, and acquired resistance through mutational changes or acquisition of plasmids conferring resistance genes^12^. Empiric therapy for individual patients based on rigorous knowledge of likely pathogens and local antibiograms should be re-assessed as soon as definitive susceptibility patterns are available which enable the selection of the optimal antimicrobial agent(s) and escalation or de-escalation of therapy^13^.

Appropriate antimicrobial treatment requires precise data of bacterial susceptibility, and the Study for Monitoring Antimicrobial Resistance Trends (SMART) integrates healthcare and pharmaceutical sectors to fill this urgent knowledge gap^14^. Initiated in 2002, this surveillance study, one of the largest and longest running in the world, includes over 200 sites from more than 60 countries. This program is designed to monitor the in vitro susceptibility of clinical Gram-negative bacterial isolates to antimicrobials in complicated respiratory, urinary, and abdominal infections worldwide; to identify early changes in resistance patterns of community or hospital-acquired organisms, including those that produce extended spectrum beta-lactamases (ESBLs), and to facilitate centralized molecular characterization of resistant bacterial isolates to better understand the mechanism of resistance.

Here, we present the first analysis of the data from a nested Canadian cohort of the SMART program which documents the antimicrobial resistance of Gram-negative infections in health institutions over the preceding 5 years according to hospital unit and infection site. This dataset provides a foundation for the more detailed exploration of precision therapeutics and antibiotic stewardship in this setting.

## Results

### Sources and frequencies of Gram-negative isolates

A total of 7180 Gram-negative isolates from adult patients were reported by Canadian hospitals to the SMART program from 2016 to 2020 (range 1300–1640 per annum) as shown in Supplementary Table 1. Of these, 327 (4.5%) lacked information on the hospital unit, the site of infection or both and were excluded from further analysis. Of the remaining 6853 isolates analyzed here, 3036 (44%) were from general medical units, 1227 (18%) from general surgical units, 1530 (22%) from intensive care units (ICU), and 1060 (15%) from emergency rooms (ER). The sources of isolates were respiratory tract infection (RTI) in 2437 (36%) cases, intra-abdominal infection (IAI) in 1744 (25%), urinary tract infection (UTI) in 1665 (24%) and bloodstream or cardiovascular system infection (CVS) in 1007 (15%). There was a significant association (p < 0.001) between hospital ward and source of isolates from 2018 to 2020 since isolates from CVS sources were not included in prior years, but there were no consistent trends in the frequencies of isolates from the selected infections between the study years.

**Figure 1:**
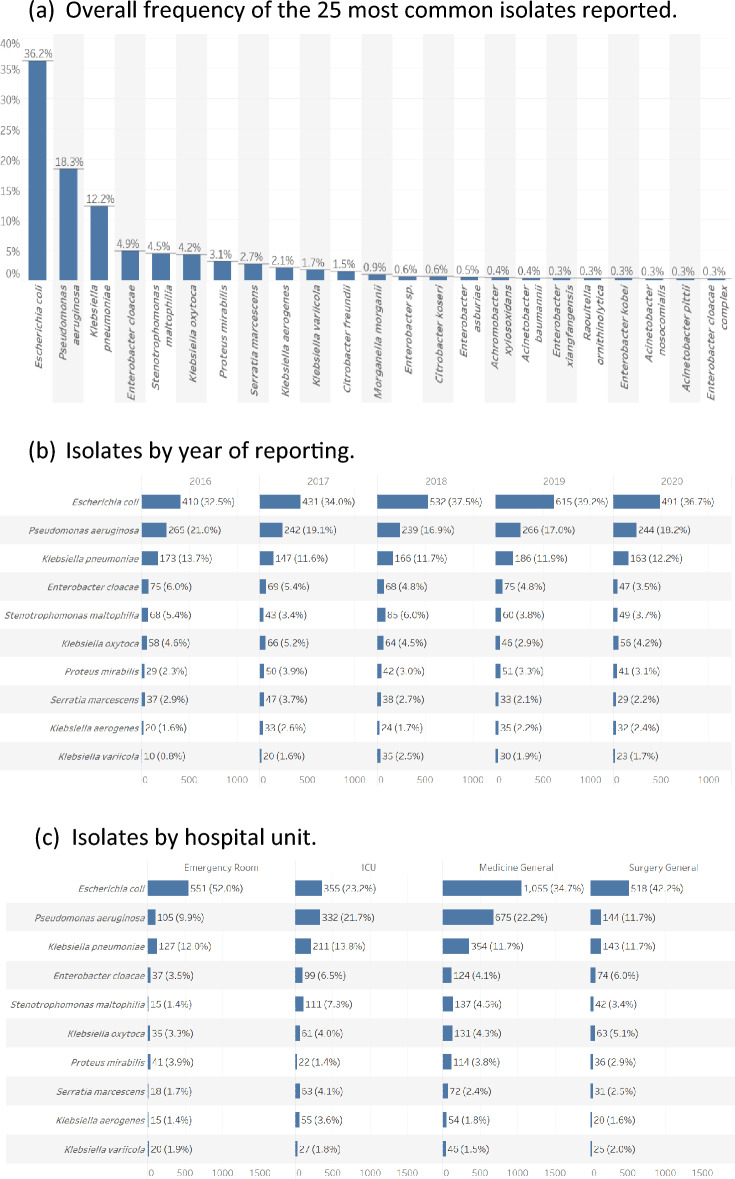

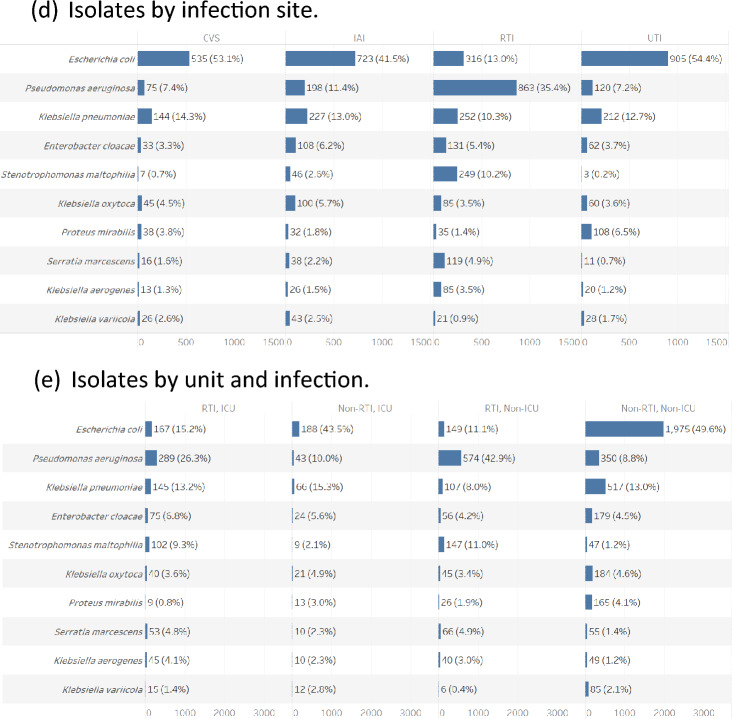
Frequency of the most common Gram-negative isolates reported to the SMART registry; (**a**) overall, (**b**) by year, (**c**) by hospital unit, (**d**) by infection site and (**e**) by unit and infection.

The most common Gram-negative isolates reported are shown overall and by year, hospital unit and infection site in Fig. 1. *E. coli* was the most frequent isolate (n = 2479, 36%), followed by *P. aeruginosa* (n = 1256, 18%), *K. pneumoniae* (n = 835, 12%), *E. cloacae* (n = 334, 5%), *S. maltophilia* (n = 305, 4%) and *K. oxytoca* (n = 290, 4%). The pattern of frequency was consistent over the reporting period. *E. coli* was the most common isolate in ICU, general medical, general surgical and ER facilities (Fig. 1c; overall p < 0.001). *P. aeruginosa* was the most common RTI pathogen in both ICU (n = 289, 26.3%) and non-ICU units (n = 574, 42.9%) while *E. coli* predominated in non-RTI infections in both ICU (n = 188, 43.5%) and non-ICU (n = 1975, 49.6%) facilities (Fig. 1e; overall p < 0.001). Other common isolates from RTI included *K. pneumoniae*, *S. maltophilia*, and *S. marcescens* with *K. pneumoniae*, *E. cloacae* and *K. oxytoca* predominating from non-RTI sources.

**Figure 2:**
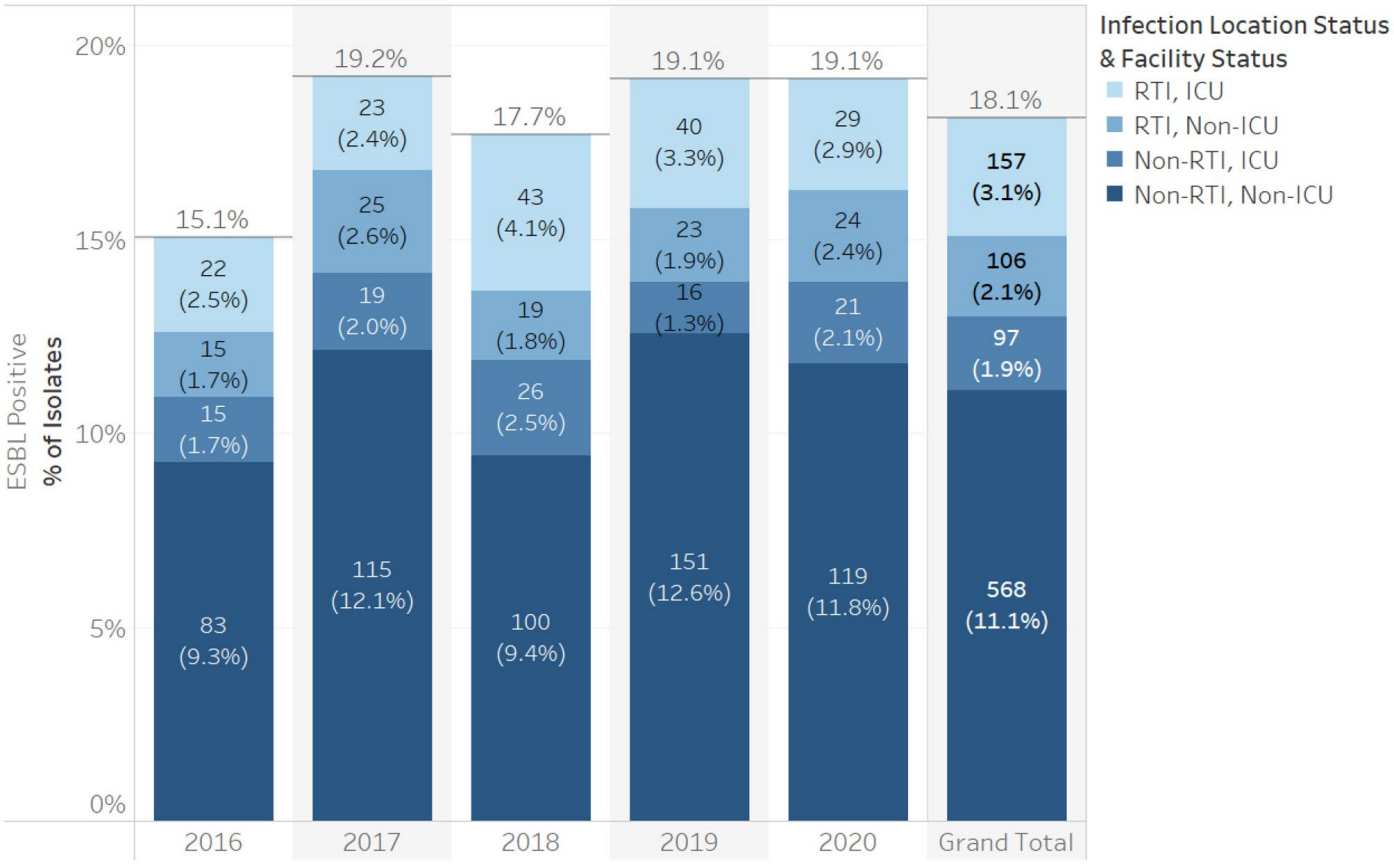
Number and proportion of Enterobacterales isolates that were ESBL positive by study year, hospital unit and infection site (RTI, respiratory tract infection).

Of the Enterobacterales species isolates analyzed in this data set, 18.1% (928/5115) were ESBL positive with no evident trend in annual frequency (annual range: 15.1–19.2%, p = 0.091) or by hospital unit or infection site (Supplementary Table 2, Fig. 2). The proportion of ESBL positivity was higher in isolates from ICU facilities (25%, 254/1017) compared with non-ICU facilities (16%, 674/4098) (p < 0.001) and in isolates from RTI (22%, 263/1199) compared with non-RTI sources (17%, 665/3916) (p < 0.001). Analysis of Enterobacterales showed a modest increase in the proportion of ESBL positive isolates over time (p = 0.042) with the following annual proportions: 2016: 15.1%; 2017: 19.2%; 2018: 17.7%; 2019: 19.1%; 2020: 19.1%.

### Susceptibility profiles

The susceptibility profiles of *P. aeruginosa* and Enterobacterales throughout the period of study are shown in Table 1. Methods employed for determining susceptibility are provided in the “Methods” section. Over 95% of *P. aeruginosa* isolates were susceptible to ceftolozane/tazobactam and colistin, while only 58–74% were susceptible to other antimicrobials including meropenem (73%), imipenem (65%), cefepime (74%), ceftazidime (72%) and piperacillin/tazobactam (68%), the antimicrobials available in Canada. Enterobacterales isolates were in general highly susceptible to meropenem (> 95%) and cefepime (85–100%), but otherwise showed great species variability. *Klebsiella, Morganella, Proteus, Raoultella, Salmonella and Serratia* were highly susceptible to all antimicrobials tested except for colistin (0%) and showed a wide range of variability to imipenem (3–100%) and levofloxacin (0–73%). *Enterobacter* isolates were normally susceptible to meropenem, imipenem and cefepime, but less susceptibility varied to ceftolozane/tazobactam (64–83%), aztreonam (64–81%), ceftazidime (54–81%), colistin, levofloxacin (0–70%) and piperacillin/tazobactam (61% to 83%).

**Table 1:**
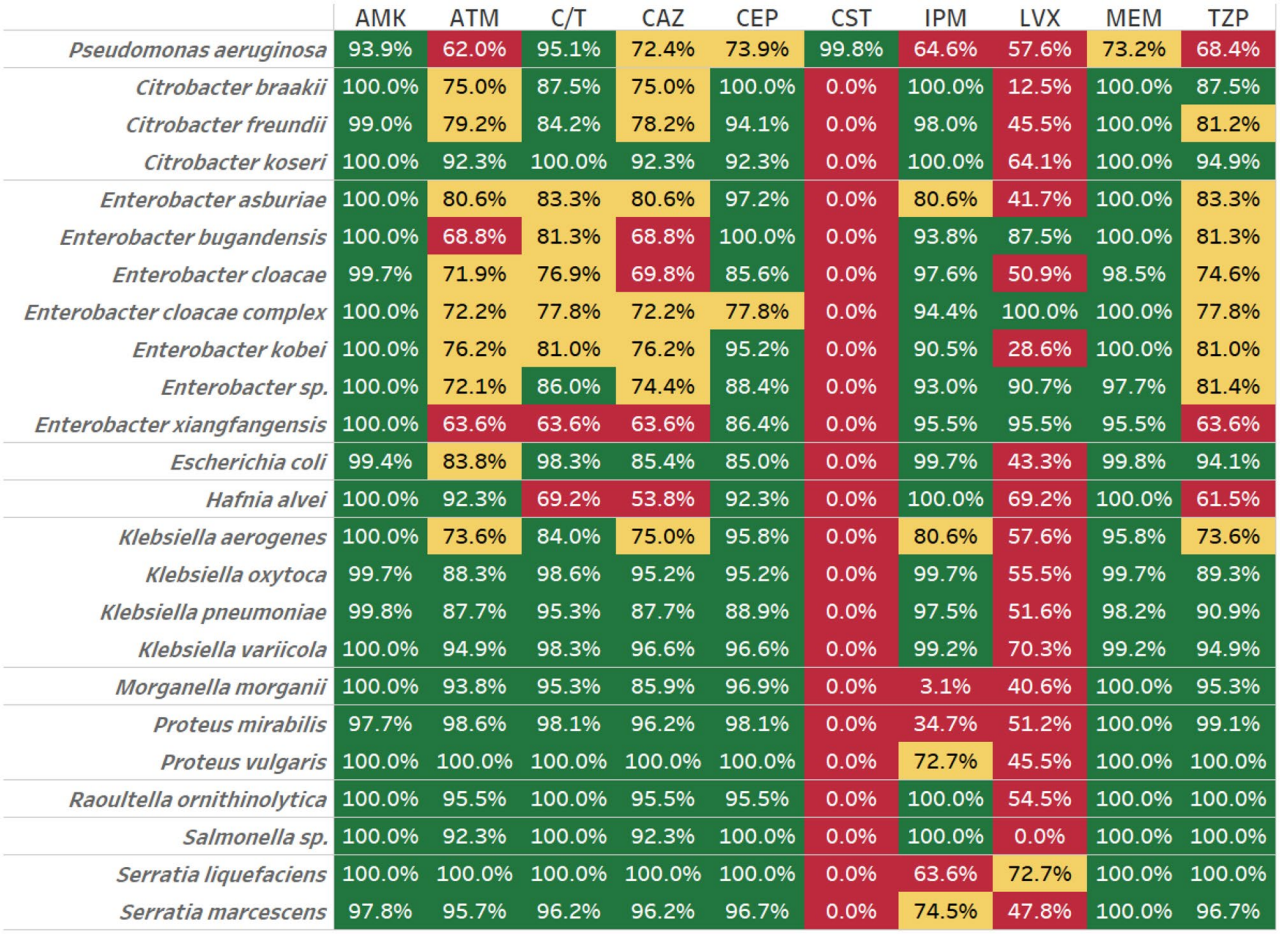
Collective antimicrobial susceptibility profile of *P. aeruginosa* and Enterobacterales isolates.

Figures show the proportion of isolates for each organism which are susceptible to the antibiotic. The antimicrobial agents tested included: amikacin (AMK), aztreonam (ATM), cefepime (CEP), cefotaxime (CTX), cefoxitin (FOX), ceftazidime (CAZ), ceftolozane/tazobactam (C/T), ceftriaxone (CRO), ciprofloxacin (CIP), colistin (CST), ertapenem (ETP), imipenem (IPM), levofloxacin (LVX), meropenem (MEM) and piperacillin/tazobactam (TZP).

Green indicates that more than 85% of isolates were susceptible, yellow that 70–85% were susceptible and red less than 70% were susceptible to the antibiotics indicated.

The susceptibility profile of ESBL-positive isolates is shown in Table 2. Between 99.5 and 100% of all taxonomic groups investigated were susceptible to meropenem (except *Pseudomonas* where 73.2% of isolates were sensitive), and *Citrobacter, Enterobacter, Escherichia, Hafnia, Klebsiella, Proteus vulgaris, Raoultella and Salmonella* isolates were fully or partially sensitive to imipenem. *Citrobacter koseri, Escherichia, Klebsiella oxytoca and variicola, Proteus, Pseudomonas, Raoultella* and *Salmonella* isolates were also highly sensitive to ceftolozane/tazobactam. The ESBL-positive isolates tested showed variable though generally lower susceptibility to aztreonam, ceftazidime, levofloxacin and piperacillin/tazobactam with the exception of *Proteus vulgaris* which was broadly sensitive to most of these agents.

**Table 2:**
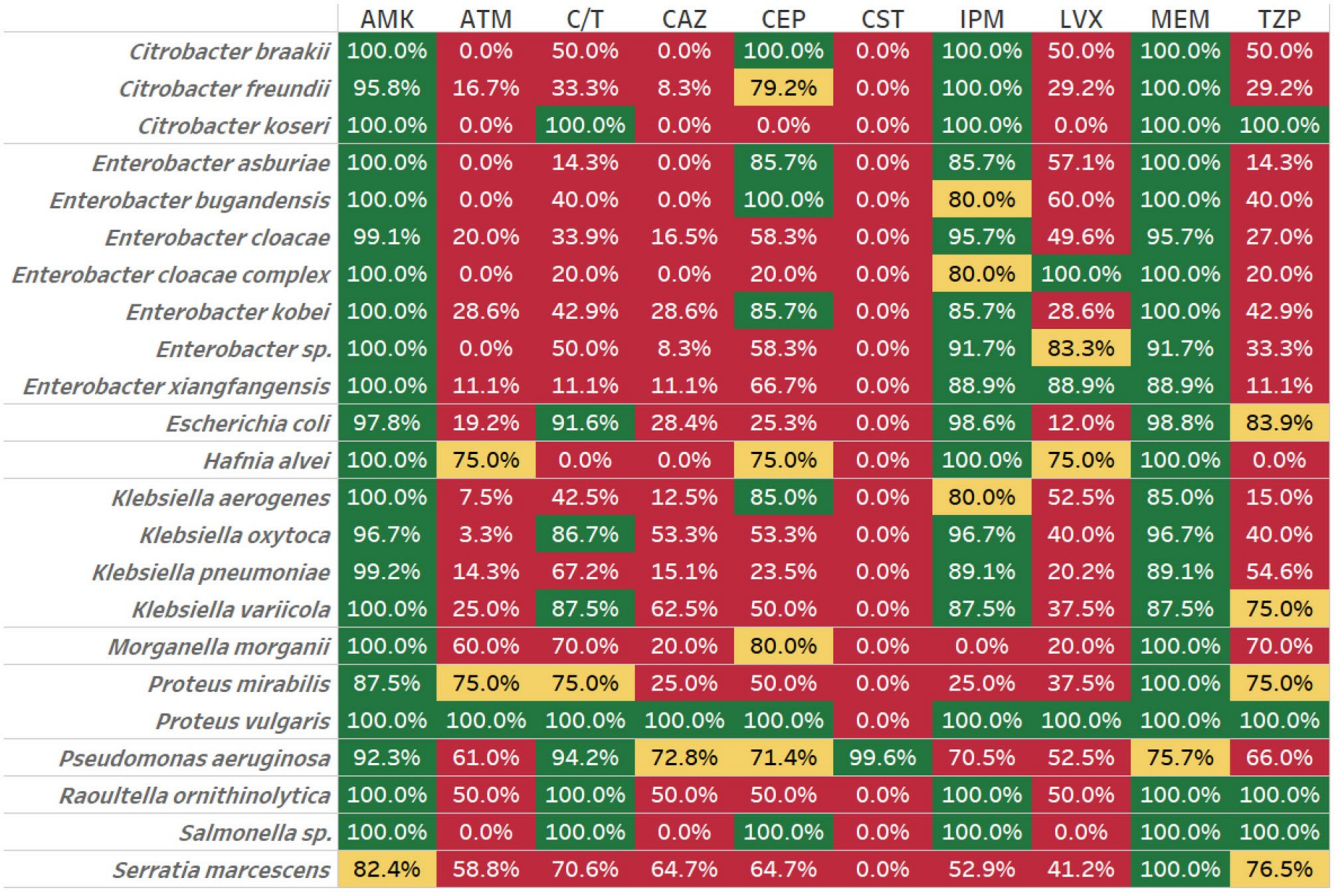
Collective antimicrobial susceptibility profile of ESBL-positive Enterobacterales isolates.

Figures show the proportion of isolates for each organism which are susceptible to the antibiotic. The antimicrobial agents tested included: amikacin (AMK), aztreonam (ATM), cefepime (CEP), cefotaxime (CTX), cefoxitin (FOX), ceftazidime (CAZ), ceftolozane/tazobactam (C/T), ceftriaxone (CRO), ciprofloxacin (CIP), colistin (CST), ertapenem (ETP), imipenem (IPM), levofloxacin (LVX), meropenem (MEM) and piperacillin/tazobactam (TZP).

Green indicates that more than 85% of isolates were susceptible, yellow that 70–85% were susceptible and red less than 70% were susceptible to the antibiotics indicated.

### Susceptibility patterns for *Pseudomonas*

#### Susceptibility of *P. aeruginosa* to first line anti-pseudomonal agents

**Figure 3:**
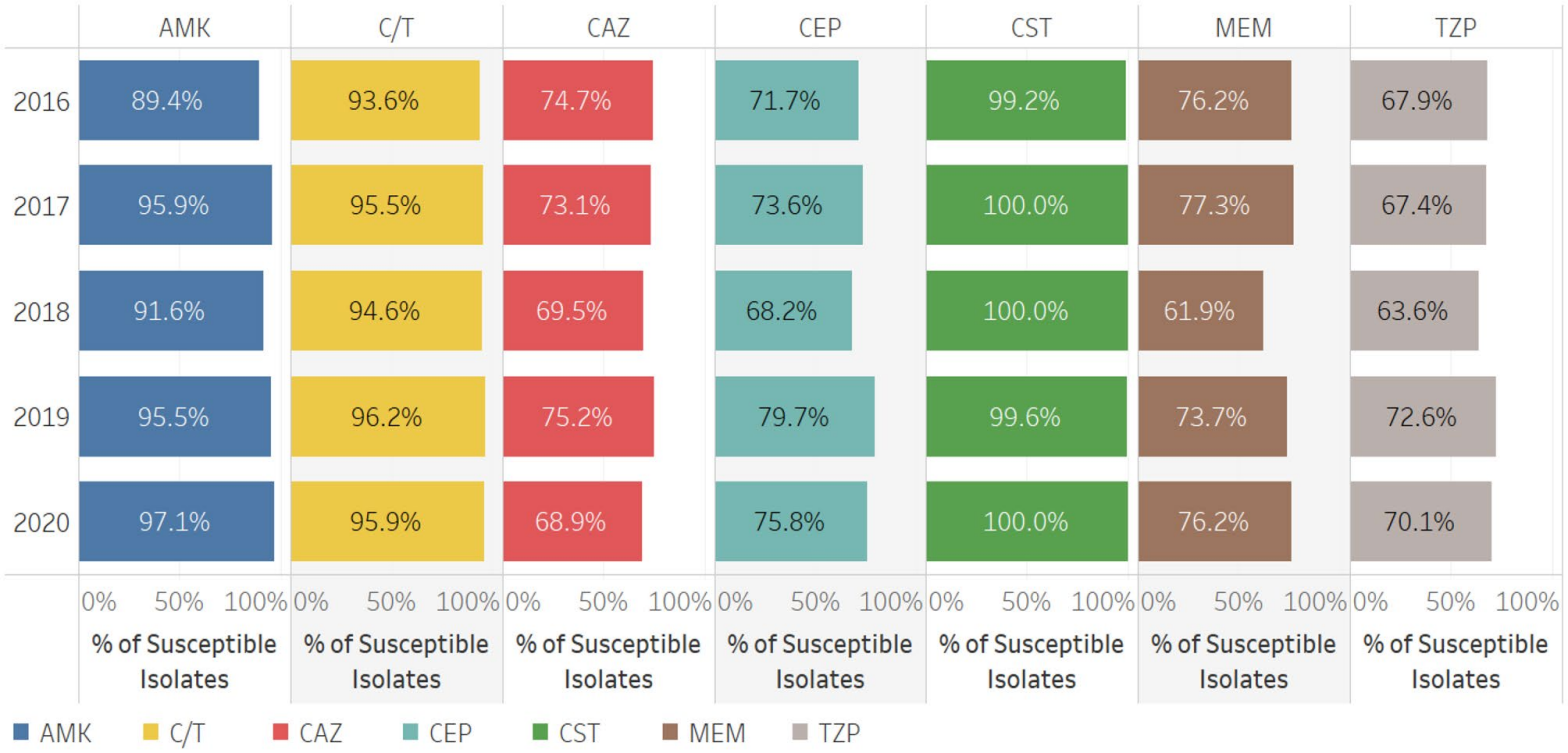
Susceptibility of *P. aeruginosa* to first line anti-pseudomonal agents from 2016 to 2020. The antimicrobial agents tested included: amikacin (AMK), aztreonam (ATM), cefepime (CEP), cefotaxime (CTX), cefoxitin (FOX), ceftazidime (CAZ), ceftolozane/tazobactam (C/T), ceftriaxone (CRO), ciprofloxacin (CIP), colistin (CST), ertapenem (ETP), imipenem (IPM), levofloxacin (LVX), meropenem (MEM) and piperacillin/tazobactam (TZP).

Antimicrobial susceptibility of *P. aeruginosa* is shown in Fig. 3. Between 94 and 96% of the isolates were susceptible to ceftolozane/tazobactam during the period of observation, comparable to the susceptibility observed to amikacin (89%-97%) and colistin (99%-100%) during this same time period. Susceptibility to cefepime, ceftazidime, meropenem and piperacillin/tazobactam was substantially lower, ranging from 60%-80% across the period of study.

#### Susceptibility of *P. aeruginosa* according to hospital unit and infection source

**Figure 4:**
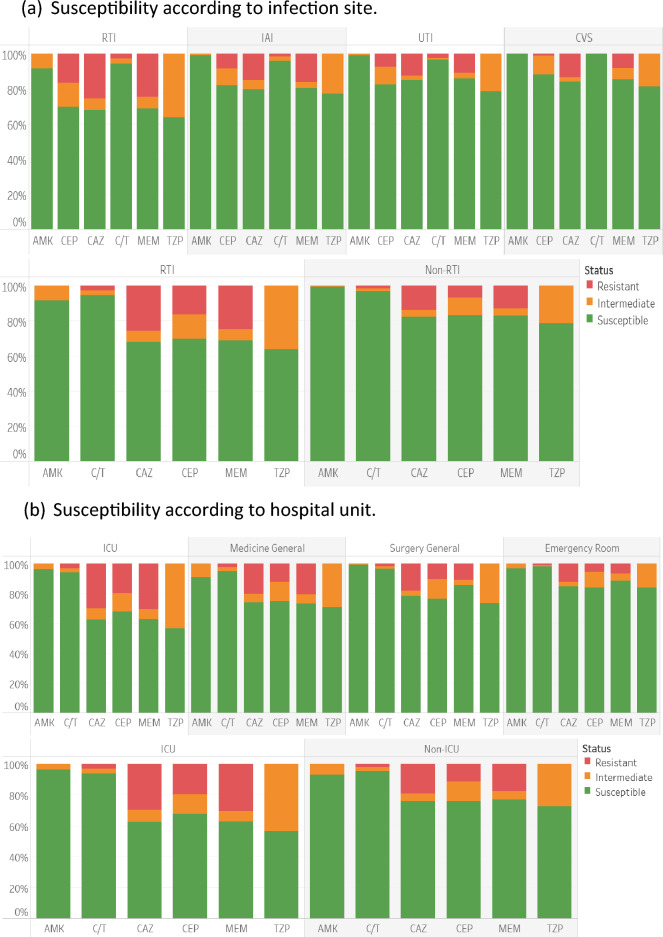
Cumulative susceptibility from 2016 to 2020 of *P. aeruginosa* according to hospital unit and infection site with “traffic-light” indication of susceptibility, intermediate and resistance. The antimicrobial agents tested included: amikacin (AMK), aztreonam (ATM), cefepime (CEP), cefotaxime (CTX), cefoxitin (FOX), ceftazidime (CAZ), ceftolozane/tazobactam (C/T), ceftriaxone (CRO), ciprofloxacin (CIP), colistin (CST), ertapenem (ETP), imipenem (IPM), levofloxacin (LVX), meropenem (MEM) and piperacillin/tazobactam (TZP).

Susceptibility of *P. aeruginosa* across the full study period and by infection site and hospital unit is shown in Fig. 4. Over 90% of isolates from all sources of infection (RTI, IAI, UTI and CVS) and hospital locations (ICU, general medicine, general surgery, emergency room) were susceptible to ceftolozane/tazobactam while they were substantially less susceptible or resistant to cefepime, ceftazidime, meropenem and piperacillin/tazobactam (Fig. 4a, b). Isolates from RTI and ICU were generally less susceptible to cefepime, ceftazidime, meropenem and piperacillin/tazobactam than those from other infections or hospital location but remained highly susceptible to ceftolozane/tazobactam (Fig. 4a, b).

### Multidrug resistance of* P. aeruginosa*

**Figure 5:**
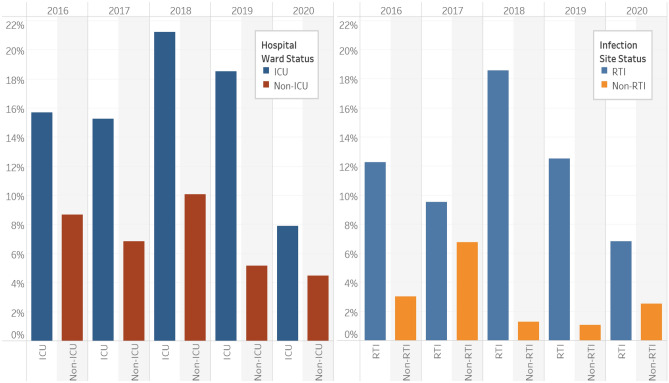
Multidrug resistance rates of *P. aeruginosa* in ICU and non-ICU settings and from respiratory tract infection (RTI) and non-RTI samples for each year of study.

As shown in Fig. 5, the frequency of multidrug resistance (MDR) in *P. aeruginosa* isolates varied by year, by hospital location and by infection source. In almost 10% of isolates MDR was observed, overall with a peak frequency of 13.4% in 2018 and a nadir of 5.8% in 2020. Multidrug resistance was twice as common overall (16% vs 8%) in isolates from ICU compared with non-ICU locations (general medicine, surgery and emergency departments) though the proportion of isolates from both locations exhibiting MDR declined by the end of the study to 8% and 5% respectively in 2020 (Fig. 5). Also, MDR was more common in isolates from RTI than non-RTI infections (13% vs 3%) the former reaching a peak of 19% in 2018 and declining to the end of the study.

**Figure 6:**
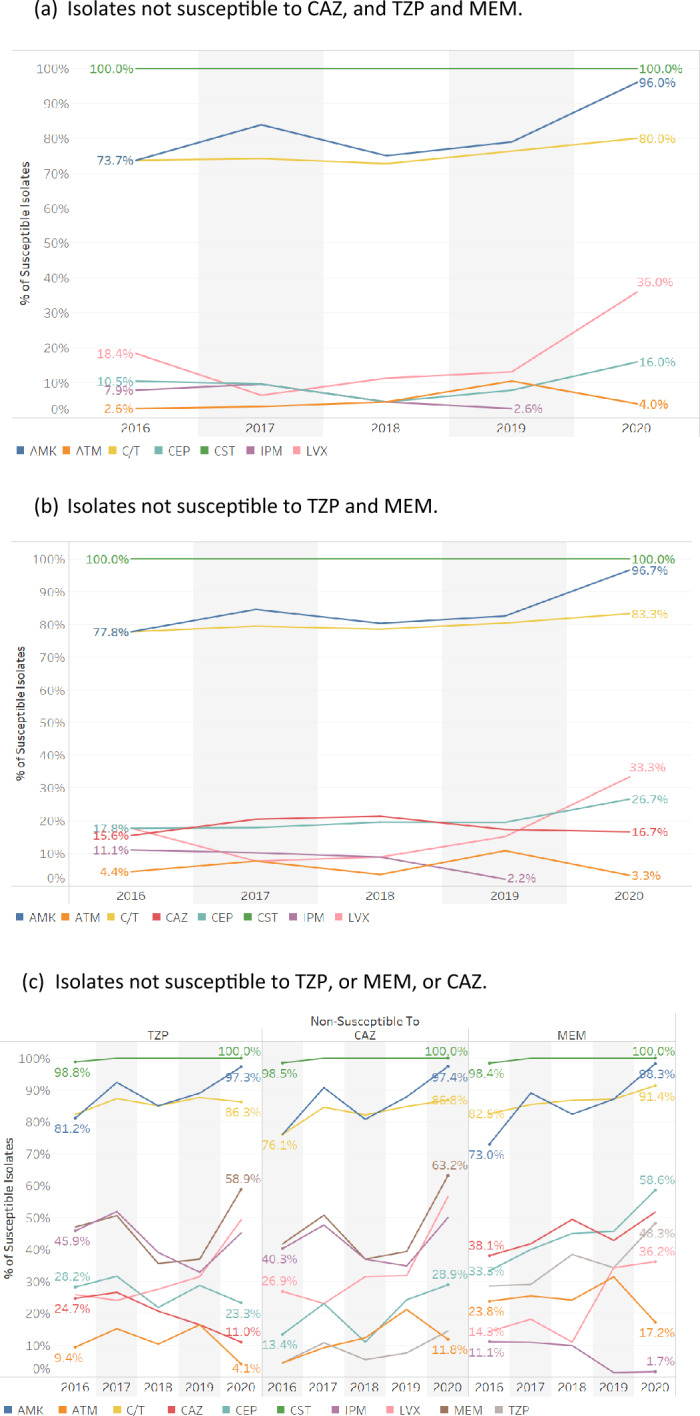
Susceptibility of *P. aeruginosa* isolates when non-susceptible (NS) to (**a**) CAZ, and TZP, and MEM, (**b**) TZP, and MEM, and (**c**) TZP, or MEM, or CAZ. The antimicrobial agents tested included: amikacin (AMK), aztreonam (ATM), cefepime (CEP), cefotaxime (CTX), cefoxitin (FOX), ceftazidime (CAZ), ceftolozane/tazobactam (C/T), ceftriaxone (CRO), ciprofloxacin (CIP), colistin (CST), ertapenem (ETP), imipenem (IPM), levofloxacin (LVX), meropenem (MEM) and piperacillin/tazobactam (TZP).

Figure 6 shows the antimicrobial susceptibility of *P. aeruginosa* isolates which were not susceptible to combinations of ceftazidime, piperacillin/tazobactam and meropenem. Figure 6a shows that 70–75% of the isolates non-susceptible to all three of these antibiotics were susceptible to ceftolozane/tazobactam over the first 4 years of study, with greater susceptibility observed in 2020 to both amikacin (96%) and to ceftolozane/tazobactam (80%). Susceptibility to cefepime remained low between 2016 and 2020. Figure 6b shows similar data for those isolates that were not susceptible to both piperacillin/tazobactam and meropenem. Between 78 and 83% of these isolates were susceptible to ceftolozane/tazobactam and 78% to 97% to amikacin. Figure 6c shows isolates that were not susceptible to the individual antimicrobials piperacillin/tazobactam, meropenem or ceftazidime. Again, between 76 and 91% of these isolates were susceptible to ceftolozane/tazobactam with susceptibility rates rising gradually from 2016 gradually to 2020. Isolates that were non-susceptible to at least one β-lactam antibiotic were susceptible only to ceftolozane/tazobactam.

## Discussion

Antimicrobial resistance is a global public health problem^15^ resulting in serious illness, prolonged hospitalization, increased mortality, and elevated healthcare costs^16, 17^. It has a profound impact on health systems in advanced nations including Canada, the U.S. and Europe^18^, as in many other regions of the world where resistance is driven by a lack of antimicrobial surveillance and antibiotic misuse^19^. Healthcare-associated infections (HAI) and AMR increase patient morbidity mortality and healthcare costs^20^. Healthcare-associated infections occur in approximately 8% of hospitalized patients in Canada and in a broad range (7–50%) in adult care units or ICU settings in other advanced economies^20–22^. While the rise in infection rate may be slowing, infections caused by antimicrobial resistant organisms continue to increase^22^. Precise knowledge of microbial susceptibility is therefore vital to enable the empiric selection of pathogen-specific antimicrobial therapy, and to adjust this quickly and effectively when required.

The SMART program provides a powerful international platform to monitor the frequency, characteristics, dynamic and geospatial trends of antimicrobial resistance across countries, hospitals, treatment facilities and sites of infection. This nested Canadian cohort provides robust adult data which enable longitudinal and regional mapping of AMR trends, and analysis of antimicrobial susceptibilities of individual isolates by hospital unit and infection site. The data shown highlight the frequency and site of the Gram-negative organisms encountered in adult patients in recent Canadian hospital practice and confirm their susceptibility profiles to current antimicrobial agents. *E. coli*, the most common isolate from all units including ICU, general medical, general surgical and ER facilities, predominated in isolates from abdominal, urinary tract and cardiovascular sources while *P. aeruginosa*, the second most prevalent organism, predominated in respiratory isolates. Deeper analysis showed that *E. coli* was the most common isolate in non-respiratory infections in both ICU (43%) and non-ICU settings (50%), while *P. aeruginosa* predominated in RTI in both these units (26% and 43% respectively). Other Critical Priority organisms including *A. baumannii* that were identified by the WHO in its 2017 report as major threats to optimal care were identified in less than 2% of isolates.

The collective susceptibility profile provides guidance for empirical treatment of the most common Gram-negative infections in adults in Canadian hospitals. Over 95% of *P. aeruginosa* isolates were susceptible to ceftolozane/tazobactam while the susceptibilities of Enterobacterales isolates were more variable. *Klebsiella, Morganella, Proteus, Raoultella, Salmonella and Serratia* were susceptible to most antimicrobials tested. *Enterobacter* isolates were normally susceptible to meropenem, imipenem and cefepime, and *E. coli* and *Hafnia* showed more variable susceptibility to many of these agents. These findings are broadly consistent with the results of both the international report from the SMART database^23^ and the Canadian CANWARD program^24–28^. The latter parallels many aspects of this nested Canadian SMART cohort in both content and intent, linking from 10 to 15 hospitals across the country and accumulating isolates from medical and surgical wards, emergency and intensive care units but also from hospital clinics. Data from these sources enable validation of the results reported here. *E. coli*, *P. aeruginosa* and *K. pneumoniae* were the most prevalent Gram-negative organisms overall in these registries, with *Pseudomonas* and *Klebsiella* predominating among ICU respiratory pathogens. Collective susceptibility profiles showed ceftolozane/tazobactam and meropenem to provide the most reliable in vitro activity against *P. aeruginosa* and Enterobacterales compared with other β-lactam antibiotics, and to offer in vitro coverage in over two-thirds of the resistant pathogens^23^. Cross-referencing of such resources is particularly important to ensure validation and to guide clinical practice in view of the relative paucity of critical information in the current literature and ongoing surveillance and reporting is required to ensure that any changes in these susceptibility profiles are noted to further direct antimicrobial stewardship and drug selection.

Production of extended-spectrum beta-lactamase (ESBL) by Enterobacterales and *P. aeruginosa* is a serious and growing clinical problem, with increased virulence potential and important disease burden^29–31^. The relatively high rates of ESBL positive Enterobacterales in Canadian institutions reported both here and in the CANWARD study are concerning^32^. They may reflect both the increasing international trend over time and the high mobility of the Canadian population^33–36^. The collective susceptibility profiles of *P. aeruginosa* and Enterobacterales confirmed the therapeutic challenge. While all organisms show high rates of susceptibility to amikacin, the response to other antimicrobial agents is variable and resistance is common. *P. aeruginosa* isolates were almost fully susceptible to amikacin, colistin and ceftolozane/tazobactam, but less susceptible to other first-line beta-lactams with intermediate susceptibility to meropenem and the 3rd and 4th generation cephalosporins ceftazidime and cefepime, and were resistant to aztreonam, imipenem, levofloxacin and piperacillin/tazobactam.

Delay in administering appropriate antimicrobial therapy has serious clinical consequences including increased mortality, hospital stay and healthcare costs^37, 38^. Accurate susceptibility information is therefore critical to guide the selection of first-line antimicrobials and to inform early switching when there is poor clinical response to first-line treatment^39^. The data presented here demonstrating non-susceptibility among the first-line beta lactams suggests it is not helpful to switch among these agents if there is no initial response. And while susceptibility is high for both amikacin and ceftolozane/tazobactam, concerns over aminoglycoside nephrotoxicity limit use of the former agent.

This study has certain limitations including selection bias, information bias and confounding which are inherent to observational design. To minimize selection bias, the study included first samples from adult patients at multiple participating hospitals across Canada, though the potential for time-varying differences in patient referral, case mix, unit services and care patterns remain. Further, sample sizes were defined for each category of infection and single isolates from sequential patients were reported within each category. While information bias may occur from many sources, stringent efforts have also been made to reduce this, in particular by examining trends within sites of infection. Location of care was consolidated into four categories of medical, surgical, ICU and emergency room facilities to reduce the variable nomenclature and diversity of case mix which differ by institution, and the site of infection was restricted to common sources seen in these settings. Notably, COVID-19 may have influenced the clinical case mix and distribution of patients within these settings during 2019–2020; however, this impact has not been thoroughly examined. Diagnostic microbiology procedures are standardized according to the Clinical Laboratory Standards Institute (CLSI) guidelines^40^ though variability in certain procedures and definition of criteria (e.g. Extended-spectrum beta-lactamase status) remains and re-testing is performed at a centralized location to ensure standardized reporting. However, and importantly, the microbiological data reported are not accompanied by clinical observations, treatments, or outcomes, thereby narrowing the interpretations which can be drawn from this data set.

The rising frequency, mortality and economic costs of AMR^41^ underscore the urgency of incorporating precision diagnostics and therapeutics within carefully-structured integrated clinical practice guidelines^39^. Vital to this approach is the rapid availability of the information required for the knowledgeable selection of first-line therapy. The SMART program offers a novel platform to begin to address this problem. First, by providing detailed information on microbial isolates and susceptibilities by institution, hospital unit and site of infection, it serves to inform local practice and evaluate national trends over time. Further, these data may be combined with clinical prediction rules^42^, genomic data^43^ and artificial intelligence programs^44–47^ to provide mobile expert antimicrobial stewardship applications^48^ that can be incorporated into treatment policies to refine care. Such comprehensive data, complementary to the public surveillance program, would provide a unique foundation for both optimal patient care and structured research in a challenging field of modern medicine.

## Methods

### Study design and ethics approval

This real-world nested cohort study examined the susceptibility patterns of Gram-negative bacilli in Canadian hospitals during the past 5 years (January 1, 2016 to December 31, 2020 inclusive). The study design complied with regulations for observational healthcare research in Canada as documented by Article 2.4 of the Tri-Council Policy Statement for Ethical Conduct for Research Involving Humans. This specifies that Research Ethics Board review is not required for research that relies exclusively on secondary use of anonymous information, or anonymous human biological materials, so long as the process of data linkage or recording or dissemination of results does not generate identifiable information. Approval for use of the anonymized data was obtained from the Global Study for Monitoring Antimicrobial Resistance and Trends (SMART) coordinated by Merck & Co., Inc., Kenilworth, NJ, USA. While both pediatric and adult patients were included, only the latter are reported here. All research reported here was performed in accordance with the Declaration of Helsinki and the Canadian Tri-Council Policy Statement for Ethical Conduct for Research Involving Humans (2018).

### Clinical samples and testing

Eight hospitals from principal health regions of Canada (Vancouver, British Columbia; Edmonton, Alberta; Saskatoon, Saskatchewan; Winnipeg, Manitoba; Montreal, Quebec; Toronto, Ontario; Trois Rivieres, Quebec; St. John, New Brunswick) participated in this program. To ensure uniformity and minimize selection bias, each was asked to submit the first Gram-negative isolate from approximately 250 sequentially infected patients per year, based on the specimen type criteria. For the years 2016 and 2017, the specimen type collection requirements consisted of 100 lower respiratory tract infections (RTI), 50 urinary tract infections (UTI) and 100 intra-abdominal infections (IAI). For 2018–2020, these were changed to 100 lower RTI, 50 UTI, 50 IAI and 50 bloodstream infections (CVS). Susceptibility testing was performed at each institution according to Clinical and Laboratory Standards Institute (CLSI) guidelines^40, 49^ and specimens were forwarded to the central reference laboratory at International Health Management Associates (IHMA, SA, Schaumburg, IL, USA) with anonymized clinical demographic data including patient age, sex, length of stay, type of hospital unit, organism and infection site.

### Microbial identification and susceptibility testing

Microbial identification was confirmed at IHMA using matrix-assisted laser desorption/ionizing time of flight spectrometry (Bruker, Daltronics). Antimicrobial susceptibility testing was performed by broth microdilution methods using CLSI recommendations and categorical interpretation of susceptibility to antimicrobial agents was reported according to the CLSI M100 guidelines (Supplementary Table 3) for the respective years^50, 51^. For ceftolozane/tazobactam, *Pseudomonas aeruginosa* breakpoints were used to interpret MICs for *Acinetobacter baumannii*. The antimicrobial agents tested included: amikacin (AMK), aztreonam (ATM), cefepime (CEP), cefotaxime (CTX), cefoxitin (FOX), ceftazidime (CAZ), ceftolozane/tazobactam (C/T), ceftriaxone (CRO), ciprofloxacin (CIP), colistin (CST), ertapenem (ETP), imipenem (IPM), levofloxacin (LVX), meropenem (MEM) and piperacillin/tazobactam (TZP)^52^. Extended-spectrum beta-lactamase (ESBL) producing organisms were determined by the CLSI ESBL-phenotypic criteria for ESBL testing, defined as an MIC value > 2 mg/L for ceftriaxone. Multi-drug resistant (MDR) isolates were defined as isolates resistant to at least one antimicrobial agent from three or more of the seven different antimicrobial categories, extensively drug resistant (XDR) were non-susceptible to five or more of the antimicrobial categories, while pandrug-resistant (PDR) were resistant to all antimicrobial agents in all categories^53^.

### Data analysis

Nested Canadian cohort data from the SMART database was provided electronically by Merck Canada Inc., Kirkland, QC, Canada to Syreon Corporation, Canada, for analysis. Data quality review was performed using software packages R and Tableau (Seattle, USA) by inspection, visualization, tabulation, and other computational processes to identify discrepancies including missing data, implausible data, outliers and zero values for single-point data. Continuous variables were summarized using number of non-missing observations, mean, standard deviation (SD), median, minimum, and maximum values, and categorical variables using the number and percentage of participants belonging to each category. The significance of differences between nominal data was analyzed using the Chi-squared test. Annual proportions of isolates harbouring ESBL genes were assessed by the Cochran-Armitage test of trend. This test was performed on Enterobacterales in the entire observation period (2016–2020) stratified by either by infection site (RTI vs non-RTI) or by hospital unit (ICU vs non-ICU). Statistical significance was defined as a p value of < 0.05.

### Supplementary Information

Supplementary Tables.

## Data Availability

The datasets used and/or analyzed during the current study are available from the corresponding author on reasonable request.

## References

[1] Lonsdale DO, Lipman J (2019). Antimicrobial resistance: We must pursue a collaborative, global approach and use a "One Health" approach. Antibiotics.

[2] Spaulding CN, Klein RD, Schreiber HL, Janetka JW, Hultgren SJ (2018). Precision antimicrobial therapeutics: The path of least resistance?. NPJ Biofilms Microbiomes.

[3] Public Health Agency of Canada. *Progress Report on the 2015 Action Plan on Antimicrobial Resistance and Use*. 2018. https://www.canada.ca/content/dam/phac-aspc/documents/services/publications/drugs-health-products/progress-report-2015-federal-action-plan-antimicrobial-resistance-use/pub-eng.pdf

[4] Pan Canadian Public Health Network. *Antimicrobial Stewardship. The Communicable and Infectious Disease Steering Committee Task Group on Antimicrobial Use Stewardship. Final Report to the Public Health Network Council, 2016.* 2016. http://www.phn-rsp.ca/pubs/anstew-gestan/pdf/pub-eng.pdf

[5] Public Health Agency of Canada. *Tackling Antimicrobial Resistance and Antimicrobial Use. A Pan-Canadian Framework for Action*. Public Health Agency of Canada. https://www.canada.ca/en/health-canada/services/publications/drugs-health-products/tackling-antimicrobial-resistance-use-pan-canadian-framework-action.html 10.14745/ccdr.v43i11a01 PMC5764732 29770049

[6] Canadian Medical Association, *Association of Medical Microbiology and Infections Disease C*. Antimicrobial Resistance (AMR). 2019. https://policybase.cma.ca/en/viewer?file=%2fdocuments%2fPolicyPDF%2fPD19-08.pdf#phrase=false 10.3138/jammi.2019-08-15.en PMC9612813 36339281

[7] Canadian Institutes of Health Research. *Antimicrobial Resistance*. Government of Canada. https://cihr-irsc.gc.ca/e/40484.html

[8] World Health Organization. *Global Priority List of Antibiotic-Resistant Bacteria to Guide Research, Discovery, and Development of New Antibiotics*. https://www.who.int/medicines/publications/WHO-PPL-Short_Summary_25Feb-ET_NM_WHO.pdf

[9] CDC. *Antibiotic Resistance Threats in the United States, 2019 (2019 AR Threats Report)*. 2019. www.cdc.gov/DrugResistance/Biggest-Threats.html

[10] Lynch JP, Clark NM, Zhanel GG (2021). Escalating antimicrobial resistance among Enterobacteriaceae: Focus on carbapenemases. Expert Opin Pharmacother..

[11] Rossolini GM, Bochenska M, Fumagalli L, Dowzicky M (2021). Trends of major antimicrobial resistance phenotypes in enterobacterales and gram-negative non-fermenters from ATLAS and EARS-net surveillance systems: Italian vs. European and global data, 2008–2018. Diagn. Microbiol. Infect. Dis..

[12] Pang Z, Raudonis R, Glick BR, Lin TJ, Cheng Z (2019). Antibiotic resistance in *Pseudomonas aeruginosa*: Mechanisms and alternative therapeutic strategies. Biotechnol. Adv..

[13] Rhodes A, Evans LE, Alhazzani W (2017). Surviving Sepsis Campaign: International guidelines for management of sepsis and septic shock: 2016. Crit. Care Med..

[14] Alliance. AI. *Study for Monitoring Antimicrobial Resistance Trends (SMART)*. https://www.amrindustryalliance.org/case-study/study-for-monitoring-antimicrobial-resistance-trends-smart/

[15] Oldenkamp R, Schultsz C, Mancini E, Cappuccio A (2021). Filling the gaps in the global prevalence map of clinical antimicrobial resistance. Proc. Natl. Acad. Sci. USA.

[16] Prestinaci F, Pezzotti P, Pantosti A (2015). Antimicrobial resistance: A global multifaceted phenomenon. Pathog. Glob. Health..

[17] Dadgostar P (2019). Antimicrobial resistance: Implications and costs. Infect. Drug Resist..

[18] CDC. *Antibiotic Resistance Threats in the United States, 2019*. 2019.

[19] Chokshi A, Sifri Z, Cennimo D, Horng H (2019). Global contributors to antibiotic resistance. J. Glob. Infect. Dis..

[20] Canadian Nosocomial Infection Surveillance Program. Healthcare-associated infections and antimicrobial resistance in Canadian acute care hospitals, 2014–2018. *Can. Commun. Dis. Rep*. 2020;46(5):99–112. 10.14745/ccdr.v46i05a01 10.14745/ccdr.v46i05a01 PMC7279130 32558807

[21] Lonsdale, D., & Lipman, J. *Global Personalization of Antibiotic Therapy in Critically Ill Patients. Expert Review of Precision Medicine and Drug Development* 87–93 (Taylor & Francis Group, 2021).

[22] Mitchell R, Taylor G, Rudnick W (2019). Trends in health care-associated infections in acute care hospitals in Canada: An analysis of repeated point-prevalence surveys. CMAJ.

[23] Moise PA, Gonzalez M, Alekseeva I (2021). Collective assessment of antimicrobial susceptibility among the most common Gram-negative respiratory pathogens driving therapy in the ICU. JAC Antimicrob. Resist..

[24] Zhanel GG, Adam HJ, Baxter MR (2013). Antimicrobial susceptibility of 22746 pathogens from Canadian hospitals: Results of the CANWARD 2007–11 study. J. Antimicrob. Chemother..

[25] Denisuik AJ, Garbutt LA, Golden AR (2019). Antimicrobial-resistant pathogens in Canadian ICUs: Results of the CANWARD 2007 to 2016 study. J. Antimicrob. Chemother..

[26] Lagacé-Wiens PRS, Adam HJ, Poutanen S (2019). Trends in antimicrobial resistance over 10 years among key bacterial pathogens from Canadian hospitals: Results of the CANWARD study 2007–16. J. Antimicrob. Chemother..

[27] Zhanel GG, Adam HJ, Baxter MR (2019). 42936 pathogens from Canadian hospitals: 10 years of results (2007–16) from the CANWARD surveillance study. J. Antimicrob. Chemother..

[28] Zhanel GG, Adam HJ (2019). Ten years of the CANWARD Study (2007–16). J Antimicrob. Chemother..

[29] Cassini A, Högberg LD, Plachouras D (2019). Attributable deaths and disability-adjusted life-years caused by infections with antibiotic-resistant bacteria in the EU and the European Economic Area in 2015: A population-level modelling analysis. Lancet Infect. Dis..

[30] Gharrah MM, Mostafa El-Mahdy A, Barwa RF (2017). Association between virulence factors and extended spectrum beta-lactamase producing. Interdiscip. Perspect. Infect. Dis..

[31] Ling W, Furuya-Kanamori L, Ezure Y, Harris PNA, Paterson DL (2021). Adverse clinical outcomes associated with carbapenem-resistant. JAC Antimicrob. Resist..

[32] Karlowsky JA, Walkty A, Golden AR (2021). ESBL-positive Escherichia coli and Klebsiella pneumoniae isolates from across Canada: CANWARD surveillance study, 2007–18. J. Antimicrob. Chemother..

[33] Woerther PL, Burdet C, Chachaty E, Andremont A (2013). Trends in human fecal carriage of extended-spectrum β-lactamases in the community: Toward the globalization of CTX-M. Clin. Microbiol. Rev..

[34] Karanika S, Karantanos T, Arvanitis M, Grigoras C, Mylonakis E (2016). Fecal colonization with extended-spectrum beta-lactamase-producing Enterobacteriaceae and risk factors among healthy individuals: A systematic review and metaanalysis. Clin. Infect. Dis..

[35] Nicolas-Chanoine MH, Gruson C, Bialek-Davenet S (2013). 10-Fold increase (2006–11) in the rate of healthy subjects with extended-spectrum β-lactamase-producing *Escherichia coli* faecal carriage in a Parisian check-up centre. J. Antimicrob. Chemother..

[36] Peirano G, Gregson DB, Kuhn S, Vanderkooi OG, Nobrega DB, Pitout JDD (2017). Rates of colonization with extended-spectrum β-lactamase-producing. CMAJ Open.

[37] Bassetti M, Rello J, Blasi F, et al. Systematic review of the impact of appropriate versus inappropriate initial antibiotic therapy on outcomes of patients with severe bacterial infections. *Int J Antimicrob Agents*. 2020;56(6):106184. 10.1016/j.ijantimicag.2020.106184 10.1016/j.ijantimicag.2020.106184 33045353

[38] Zasowski EJ, Bassetti M, Blasi F (2020). A systematic review of the effect of delayed appropriate antibiotic treatment on the outcomes of patients with severe bacterial infections. Chest.

[39] Tamma, P., Aitken, S., Bonomo, R., Mathers, A., van Duin, D., & Clancy, C. *ISDA Guidance on the Treatment of Antimicrobial-Resistant Gram-Negative Infections: Version 1.0*. https://www.idsociety.org/practice-guideline/amr-guidance/10.1093/cid/ciad428 37463564

[40] Clinical and Laboratory Standards Institute. *Performance Standards for Antimicrobial Disc Susceptibility Tests. 13th ed. CLSI Standard MO2.* 2018.

[41] Council of Canadian Academies. *When Antibiotics Fail: The Expert Panel on the Potential Socio-economic Impacts of Antimicrobial Resistance in Canada.* 2019. https://cca-reports.ca/wp-content/uploads/2018/10/When-Antibiotics-Fail-1.pdf

[42] Vock I, Aguilar-Bultet L, Egli A, Tamma PD, Tschudin-Sutter S (2020). Independent, external validation of clinical prediction rules for the identification of extended-spectrum β-lactamase-producing Enterobacterales, University Hospital Basel, Switzerland, January 2010 to December 2016. Euro Surveill..

[43] MacFadden DR, Coburn B, Břinda K (2020). Using genetic distance from archived samples for the prediction of antibiotic resistance in. Antimicrob. Agents Chemother..

[44] Pascucci M, Royer G, Adamek J (2021). AI-based mobile application to fight antibiotic resistance. Nat. Commun..

[45] Fanelli U, Pappalardo M, Chinè V (2020). Role of artificial intelligence in fighting antimicrobial resistance in pediatrics. Antibiotics.

[46] Sutton RT, Pincock D, Baumgart DC, Sadowski DC, Fedorak RN, Kroeker KI (2020). An overview of clinical decision support systems: Benefits, risks, and strategies for success. NPJ Digit. Med..

[47] Bruckert S, Finzel B, Schmid U (2020). The next generation of medical decision support: A roadmap toward transparent expert companions. Front. Artif. Intell..

[48] Elligsen M, Pinto R, Leis JA, Walker SAN, Daneman N, MacFadden DR (2021). Improving Decision Making in Empiric Antibiotic Selection (IDEAS) for Gram-negative Bacteremia: A prospective clinical implementation study. Clin Infect Dis..

[49] Clinical and Laboratory Standards Institute. *M39-A4: Analysis and Presentation of Cumulative Antimicrobial Susceptibility Test Data; Approved Guideline—Fourth Edition.* 2014. https://clsi.org/

[50] Hackel MA, Tsuji M, Yamano Y, Echols R, Karlowsky JA, Sahm DF (2019). Reproducibility of broth microdilution MICs for the novel siderophore cephalosporin, cefiderocol, determined using iron-depleted cation-adjusted Mueller–Hinton broth. Diagn. Microbiol. Infect. Dis..

[51] CLSI. *Performance Standards for Antimicrobial Disc Susceptibility Tests*, 13th ed*. CLSI Standard MO2.* 2018.

[52] Antimicrobial Agents and Therapy. *AAC Abbreviations*. *ASM Journals*. https://journals.asm.org/journal/aac/abbreviations

[53] Magiorakos AP, Srinivasan A, Carey RB (2012). Multidrug-resistant, extensively drug-resistant and pandrug-resistant bacteria: An international expert proposal for interim standard definitions for acquired resistance. Clin. Microbiol. Infect..

