# Supplementary material for: A nested cohort 5-year Canadian surveillance of Gram-negative antimicrobial resistance for optimized antimicrobial therapy
Source: Sci Rep. 2023 Aug 29;13:14142. doi: 10.1038/s41598-023-40012-z (PMC10465604; doi:10.1038/s41598-023-40012-z)
Supplement: Supplementary file 1 — Supplementary Tables. [file 41598_2023_40012_MOESM1_ESM.docx]

**Supplementary Materials**

Supplementary Table 1. Gram-negative isolates reported to the SMART study database by year, hospital unit and site of infection. The sites of infection included respiratory tract infection (RTI), intra-abdominal infection (IAI), urinary tract infection (UTI) and bloodstream or cardiovascular system (CVS).


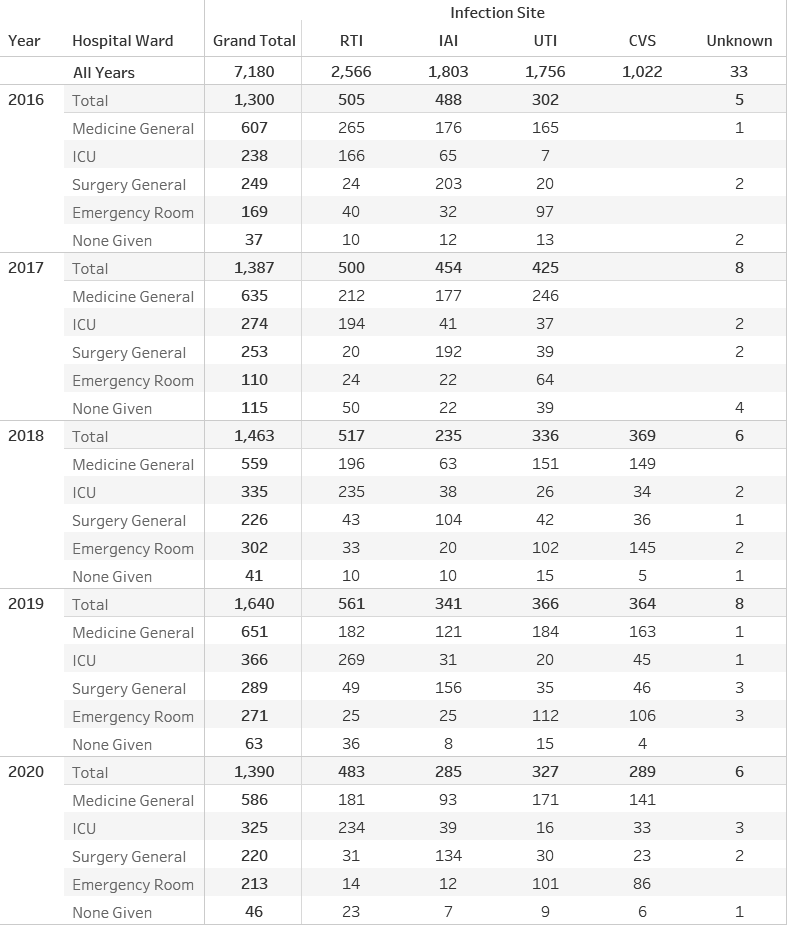


Supplementary Table 2: Enterobacterales isolates reported to the SMART study database by year, identified by ICU or non-ICU location, by respiratory tract (RTI) or non-respiratory tract (non-RTI) infection and by extended-spectrum beta-lactamase (ESBL) positivity.


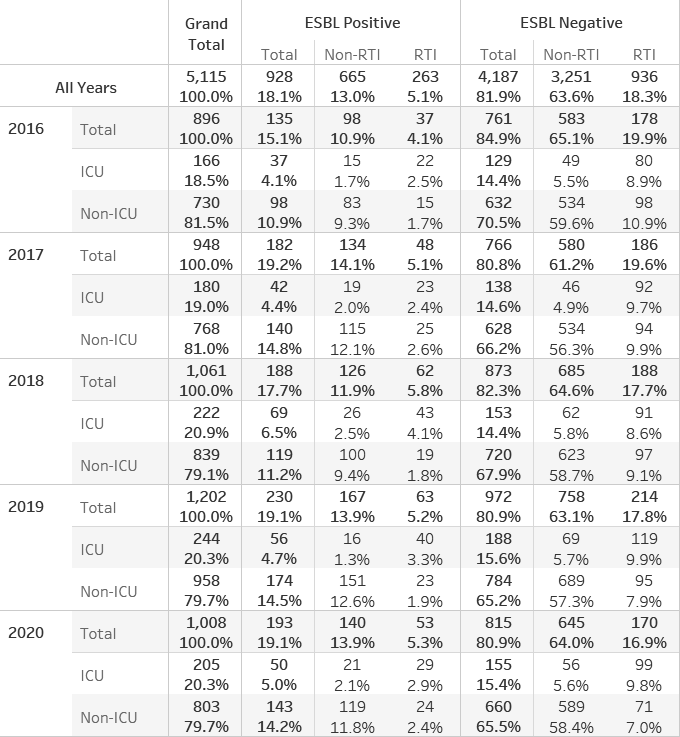


Supplementary Table 3: CLSI recommended breakpoints (Performance Standards for Antimicrobial Susceptibility Testing, M100; Malvery, PA, March 29, 2021).

| **Drug** | **Enterobacteriaceae** | | | **Pseudomonas aeruginosa** | | |
| --- | --- | --- | --- | --- | --- | --- |
|  | **Susceptible** | **Intermediate** | **Resistant** | **Susceptible** | **Intermediate** | **Resistant** |
| Amikacin | ≤16 | 32 | ≥64 | ≤16 | 32 | ≥64 |
| Azithromycin | ≤4 | 8 | ≥16 | ≤8 | 16 | ≥32 |
| Cefepime | ≤2 | 4-8 | ≥16 | ≤8 | 16 | ≥32 |
| Cefotaxime | ≤1 | 2 | ≥4 |  |  |  |
| Cefazolin | ≤8 | 16 | ≥32 |  |  |  |
| Ceftazidime | ≤4 | 8 | ≥16 | ≤8 | 16 | ≥32 |
| Ceftazidime/tazobactam | ≤2/4 | 4/4 | ≥8/4 | ≤4/4 | 8/4 | ≥16/4 |
| Ceftriaxone | ≤1 | 2 | ≥4 |  |  |  |
| Ciprofloxacin* | ≤0.25 | 0.5 | ≥1 | ≤0.5 | 1 | ≥2 |
| Colistin |  | ≤2 | ≥4 |  | ≤2 | ≥4 |
| Ertapenem | ≤0.5 | 1 | ≥2 |  |  |  |
| Imipenem | ≤1 | 2 | ≥4 | ≤2 | 4 | ≥8 |
| Levofloxacin* | ≤0.5 | 1 | ≥2 | ≤1 | 2 | ≥4 |
| Meropenem | ≤1 | 2 | ≥4 | ≤2 | 4 | ≥8 |
| Piperacillin/tazobactam | ≤16/4 | 32/4-64/4 | ≥128/4 | ≤16/4 | 32/4-64/4 | ≥128/4 |

*Breakpoints for Salmonella spp. Are ≤0.06, 0.12-0.5, ≥1 for ciprofloxacin and ≤0.12, 0.25-1 and ≥2 for levofloxacin
